# Seq2Phase: language model-based accurate prediction of client proteins in liquid–liquid phase separation

**DOI:** 10.1093/bioadv/vbad189

**Published:** 2023-12-22

**Authors:** Kazuki Miyata, Wataru Iwasaki

**Affiliations:** Department of Biological Sciences, Graduate School of Science, The University of Tokyo, Bunkyo-ku, Tokyo 113-0032, Japan; Department of Biological Sciences, Graduate School of Science, The University of Tokyo, Bunkyo-ku, Tokyo 113-0032, Japan; Department of Integrated Biosciences, Graduate School of Frontier Sciences, The University of Tokyo, Chiba 277-0882, Japan; Department of Computational Biology and Medical Sciences, Graduate School of Frontier Sciences, The University of Tokyo, Kashiwa, Chiba 277-0882, Japan; Atmosphere and Ocean Research Institute, The University of Tokyo, Kashiwa, Chiba 277-0882, Japan; Institute for Quantitative Biosciences, The University of Tokyo, Bunkyo-ku, Tokyo 113-0032, Japan; Collaborative Research Institute for Innovative Microbiology, The University of Tokyo, Bunkyo-ku, Tokyo 113-0032, Japan

## Abstract

**Motivation:**

Liquid–liquid phase separation (LLPS) enables compartmentalization in cells without biological membranes. LLPS plays essential roles in membraneless organelles such as nucleoli and p-bodies, helps regulate cellular physiology, and is linked to amyloid formation. Two types of proteins, scaffolds and clients, are involved in LLPS. However, computational methods for predicting LLPS client proteins from amino-acid sequences remain underdeveloped.

**Results:**

Here, we present Seq2Phase, an accurate predictor of LLPS client proteins. Information-rich features are extracted from amino-acid sequences by a deep-learning technique, Transformer, and fed into supervised machine learning. Predicted client proteins contained known LLPS regulators and showed localization enrichment into membraneless organelles, confirming the validity of the prediction. Feature analysis revealed that scaffolds and clients have different sequence properties and that textbook knowledge of LLPS-related proteins is biased and incomplete. Seq2Phase achieved high accuracies across human, mouse, yeast, and plant, showing that the method is not overfitted to specific species and has broad applicability. We predict that more than hundreds or thousands of LLPS client proteins remain undiscovered in each species and that Seq2Phase will advance our understanding of still enigmatic molecular and physiological bases of LLPS as well as its roles in disease.

**Availability and implementation:**

The software codes in Python underlying this article are available at https://github.com/IwasakiLab/Seq2Phase.

## 1 Introduction

Liquid–liquid phase separation (LLPS, also known as liquid–liquid phase transition) enables intracellular compartmentalization without biological membranes and regulates cellular physiology. LLPS forms membraneless organelles, such as nucleoli and p-bodies, by the spontaneous aggregation of biomolecules (Brangwynne *et al.* 2011, Kroschwald *et al.* 2015, Lin *et al.* 2015, Weber and Brangwynne 2015, Feric *et al.* 2016). LLPS is also known to be associated with the formation of amyloids, which are a cause of neurodegenerative diseases (Patel *et al.* 2015).

LLPS forms fluid assemblies known as condensates, which consist of two groups of proteins: scaffolds and clients. Scaffolds are proteins that spontaneously form condensates, and clients are proteins that are recruited into condensates but are not essential for condensation (Ditlev *et al.* 2018). Scaffolds are the main components of membrane-less organelles, and their mutations can cause diseases, such as those of FUS and TDP-43 (Patel *et al.* 2015, Mann *et al.* 2019). On the other hand, clients play a variety of roles in membraneless organelles including enzymatic reactions and signal transduction, and have been found to regulate LLPS (Mondal *et al.* 2022, Tan *et al.* 2023). In addition, clients of stress granules were suggested to be associated with neurodegenerative diseases (Patel *et al.* 2015, Markmiller *et al.* 2018). Whereas clients contain a much wider variety of proteins than scaffolds, our understanding of the molecular and physiological characteristics of condensate clients, as well as their roles in disease initiation and progression, is still severely limited.

Like the bioinformatics classics of protein localization prediction, an accurate predictor of condensate proteins is needed for systematic analysis of LLPS. First-generation LLPS predictors were built based on studies of sequence properties of scaffold proteins (Vernon and Forman-Kay 2019). General characteristics of a scaffold include intrinsically disordered and low-complexity regions (Elbaum-Garfinkle *et al.* 2015, Molliex *et al.* 2015, Nott *et al.* 2015). CatGranule uses protein length, intrinsically disordered regions, RNA binding, and Phe, Gly, Arg ratios (Bolognesi *et al.* 2016). PScore uses sp^2^ electron interaction (Vernon *et al.* 2018). Recent machine learning-based methods further improved the prediction performance (Saar *et al.* 2021, Chen *et al.* 2022, Chu *et al.* 2022). Those scaffold predictors led to the experimental identification of new condensate components.

However, sequence properties of client proteins are still poorly understood, although a high abundance of charged amino acids and low solvation energy in the aqueous phase were hypothesized to be important (Villegas and Levy 2022, Jo *et al.* 2022). It is even unclear if amino-acid sequences of client proteins share physicochemical characteristics or if individual interactions with scaffold proteins recruit clients to condensates (Jo *et al.* 2022). Last but not least, there have not been computational predictors for clients, which occupy a substantial part of LLPS condensates.

Deep learning-based methods are effective for classification problems where such knowledge is not available in advance. Transformer, a deep-learning-based method that embeds natural language into vectors, was recently adopted for various classification problems in biology and has shown promising performances (Vaswani *et al.* 2017, Hou *et al.* 2022). Such language model-based tools include ProtTrans and ESM-2, which represent amino-acid sequences by vectors that can be used as inputs for various machine-learning methods (Elnaggar *et al.* 2022, Lin *et al.* 2023).

Here, we developed Seq2Phase, the first computational predictor of LLPS client proteins to the best of our knowledge. By combining ProtTrans (Elnaggar *et al.* 2022) and an ensemble model of four machine learning models, support vector machine (SVM), random forest (RF), histogram-based gradient boosting classification tree (HGBC), and neural network (NN), accurate predictions were achieved with ROC AUC = 0.86. Predicted client proteins contained known LLPS regulators and showed localization enrichment into membraneless organelles, confirming the validity of the prediction. Feature analysis revealed that scaffolds and clients have different sequence properties and that textbook knowledge of LLPS-related proteins is biased and incomplete. Seq2Phase achieved high accuracies across human, mouse, yeast, and plant, showing that the method is not overfitted to specific species and has broad applicability. We predict that more than hundreds or thousands of LLPS client proteins remain undiscovered in each species and that Seq2Phase will advance our understanding of still enigmatic molecular and physiological bases of LLPS as well as its roles in disease.

## 2 Methods

### 2.1 Data construction

Amino-acid sequences of *Homo sapiens* (20398 sequences) and *Saccharomyces cerevisiae* strain ATCC 204508/S288c (6727 sequences) proteomes were downloaded from Swiss-Prot on September 16, 2022 (Bateman *et al.* 2022). UniProt IDs of scaffolds, clients, and regulators were obtained from DrLLPS v1.0 (Ning *et al.* 2020). CD-HIT v. 4.8.1 was used for clustering with parameters set to *-c 0.5 -n 2* (Li and Godzik 2006, Fu *et al.* 2012).

### 2.2 Sequence property analysis

The proportion of intrinsically disordered regions was obtained by calculating the degrees of disorder of each amino acid using IUPred3 long disorder with default parameters and averaging them across the whole protein length (Erdős *et al.* 2021). Hydrophobicity was calculated by averaging the Kyte-Doolittle scale for each amino acid over the entire length of the protein (Kyte and Doolittle 1982). The proportion of charged amino acids was calculated as the proportion of Asp, Glu, Lys, or Arg. The PScore was determined based on the per-protein score (Vernon *et al.* 2018). The low-complexity regions were calculated using the SEG algorithm with default parameters (Wootton and Federhen 1993).

### 2.3 Embedding

Amino acid sequences were embedded into vectors using ProtTrans and ESM2 (Elnaggar *et al.* 2022, Lin *et al.* 2023). ProtTrans was run on bio_embeddings (v0.2.2), and the model was ProtTransT5XLU50Embedder (half_precision_model=False) (Dallago *et al.* 2021). The version of ESM was 2.0.0, and the model used was esm.pretrained.esm2_t36_3B_UR50D. To obtain per-protein vectors, the vectors obtained per residue were averaged over the entire protein length. To run on the GPU (NVIDIA A100), we used pytorch1.12.1+cu116 (Paszke *et al.* 2019).

### 2.4 Machine learning

To compare the performance of various machine learning models in predicting clients, a 5-fold cross-validation was carried out utilizing the StratifiedKFold method from scikit-learn, set with n_splits = 5 and shuffle=True. We limited our dataset to sequences of fewer than 1000 amino acids for both client and non-LLPS classes.

Two embedding techniques were used: the ProtTrans T5XLU50 model (PT-T5XLU50) and the ESM2 3B model (ESM2-3B). To enable fair comparison, ESM2-3B embeddings were dimensionally reduced using principal component analysis (PCA), which was fitted only on the training data. This reduced its dimensionality to 1024, the same as that of PT-T5XLU50. Both the dimension-reduced and original ESM2-3B embeddings were used in the study, alongside PT-T5XLU50.

Due to imbalances in the numbers of client and non-LLPS proteins in the training datasets, downsampling by a random approach and Tomek links was also conducted. The test data retained its original imbalance.

The ML methods used were RF, SVM, HGBC, and NN (Cortes and Vapnik 1995, Breiman 2001, Ke *et al.* 2017). RF, SVM, and HGBC were computed with scikit-learn 0.23.2, and NN with Pytorch 1.10.0+cu102 (Pedregosa *et al.* 2011, Paszke *et al.* 2019). A two-layer forward neural network was used for NN: nn. Sequential**(**nn.Linear(1024, 32), nn. Dropout(0.25), nn. ReLU(), Linear(32,2)). Adam(lr = 0.01) was used as the optimizer, and the loss function was the cross-entropy loss. The computation was stopped if the loss function did not decrease for ten epochs.

For hyperparameter optimization, we performed a grid search on the training data for each of the machine learning models using scikit-learn GridSearchCV. The explored hyperparameters for RF included “max_depth” with values [5, 10, 15, 20] and “max_features” with options [“log2,” “sqrt,” None]. SVM was subjected to a grid search across “C” values [1, 10, 100], “kernel” types [“poly,” “rbf,” “sigmoid”], and “gamma” settings [“scale,” “auto”]. For HGBC, we examined “learning_rate” [0.05, 0.1, 0.2], “max_leaf_nodes” [15, 31, 63], and “min_samples_leaf” [20, 40, 80]. In the case of NN, learning rates (“lr”) [0.01, 0.02, 0.03, 0.04, 0.05] were explored. Following the identification of optimal hyperparameters, the models were then trained on the entire training dataset.

### 2.5 Model stacking for ensemble learning

The chosen hyperparameters for each of RF, HGBC, SVM, and NN were used (RF with max_depth = 20, max_features=“sqrt,” class_weight=“balanced,” and n_estimators = 200, HGBC with learning_rate = 0.1, max_leaf_nodes = 63, min_samples_leaf = 80, and class_weight=“balanced,” SVM with class_weight=“balanced,” probability=True, gamma=“auto,” and NN with lr = 0.01). Logistic regression was conducted by scikit-learn StackingClassifier with default settings. This model was compared with an SVM utilizing the selected hyperparameters, using 10-fold cross-validation. No downsampling was conducted.

### 2.6 Scaffold prediction

For scaffold prediction, we optimized the dimensionality of input vectors and hyperparameters for each machine learning model constituting the stacked model. The dimensionality of inputs for each model was reduced using PCA to dimensions of 128, 64, 32, 16, 8, and 4. Subsequently, hyperparameter tuning was conducted for each dimension using GridSearchCV. The explored hyperparameters for RF included “max_depth” with values [5, 10, 15, 20] and “max_features” with options [“log2,” “sqrt,” None]. SVM was subjected to a grid search across “C” values [1, 10], “kernel” types [“rbf,” “sigmoid”], and “gamma” settings [“scale,” “auto”]. For HGBC, we examined “learning_rate” [0.05, 0.1, 0.2], “max_leaf_nodes” [15, 31, 63], and “min_samples_leaf” [20, 40, 80]. In the case of NN, learning rates (“lr”) [0.01, 0.02, 0.03, 0.04, 0.05] were explored. Performance evaluation was based on the average PR-AUC of a 5-fold cross-validation. Both PCA fitting and grid search were executed using the training dataset.

As a result, for the classification of scaffold versus non-LLPS, the optimal parameters were set as: NN at 128 dimensions with lr = 0.05; RF at 128 dimensions with max_depth = 5 and max_features=“log2”; SVM at 128 dimensions with C = 1, gamma=“scale,” and kernel=“rbf”; and HGBC at 64 dimensions with learning_rate = 0.1, max_leaf_node = 31, and min_samples_leaf = 40. For the classification of scaffold versus client, the optimal parameters were: NN at 128 dimensions with lr = 0.05; RF at 128 dimensions with max_depth = 5 and max_features=None; SVM at 32 dimensions with C = 1, gamma=“scale,” and kernel=“rbf”; and HGBC at 64 dimensions with learning_rate = 0.05, max_leaf_node = 15, and min_samples_leaf = 20.

### 2.7 GO enrichment analysis

GO enrichment analysis was performed using GOA tools v.1.2.4 (Klopfenstein *et al.* 2018). The ontology file go-basic.obo was downloaded and the human GO annotation file goa_human.gaf was downloaded on 5 December 2022, from http://geneontology.org (Ashburner *et al.* 2000, Gene Ontology Consortium 2021). Only cellular-component terms were used in this study. Fisher’s exact probability test was performed with multiple testing corrections to control false discovery rates using the Benjamini–Hochberg method (Fisher 1935, Benjamini and Hochberg 1995). Odds ratios were calculated as follows: (# of Class A with a GO/# of Class A without a GO)/(# of Class B with a GO/# of Class B without a GO).

### 2.8 UMAP

The vectors obtained per protein were visualized by UMAP using Python (v0.5.3) (McInnes *et al.* 2018). The parameters used were random_state = 0, n_neighbors = 10, and min_dist = 0.7.

### 2.9 Inter-species client prediction

Amino-acid sequences of *Mus musculus* and *Arabidopsis thaliana* proteomes were downloaded from Swiss-Prot on 22 December 2022. Sequence clustering was performed as previously. To remove protein sequences similar to those of *H.sapiens*, DIAMOND (v2.0.14) in—*ultra-sensitive* mode was used at a threshold of ppos > 40 (Buchfink *et al.* 2021).

### 2.10 Region-wise prediction

We derived vectors per amino acid using PT-T5XLU50 from the full length of the amino-acid sequences. We used the sliding-window technique to calculate the average vectors. The window size was set to 100 amino acids, while it was shortened at the ends of the sequences to make the vector length the same as the sequence length. Seq2Phase was then used to compute a client score for each position. We designated positions that scored 0.5 or above as client-like regions.

## 3 Results

### 3.1 Dataset construction

Amino-acid sequences of the proteomes of *H.sapiens* and *S.cerevisiae* were downloaded from Swiss-Prot (Bateman *et al.* 2022). Scaffold and client proteins of both species were downloaded from DrLLPS, a comprehensive database of LLPS-related proteins in literature (Ning *et al.* 2020). DrLLPS also contained data on regulator proteins, which are known to interact with and affect condensates.

**Figure 1.**
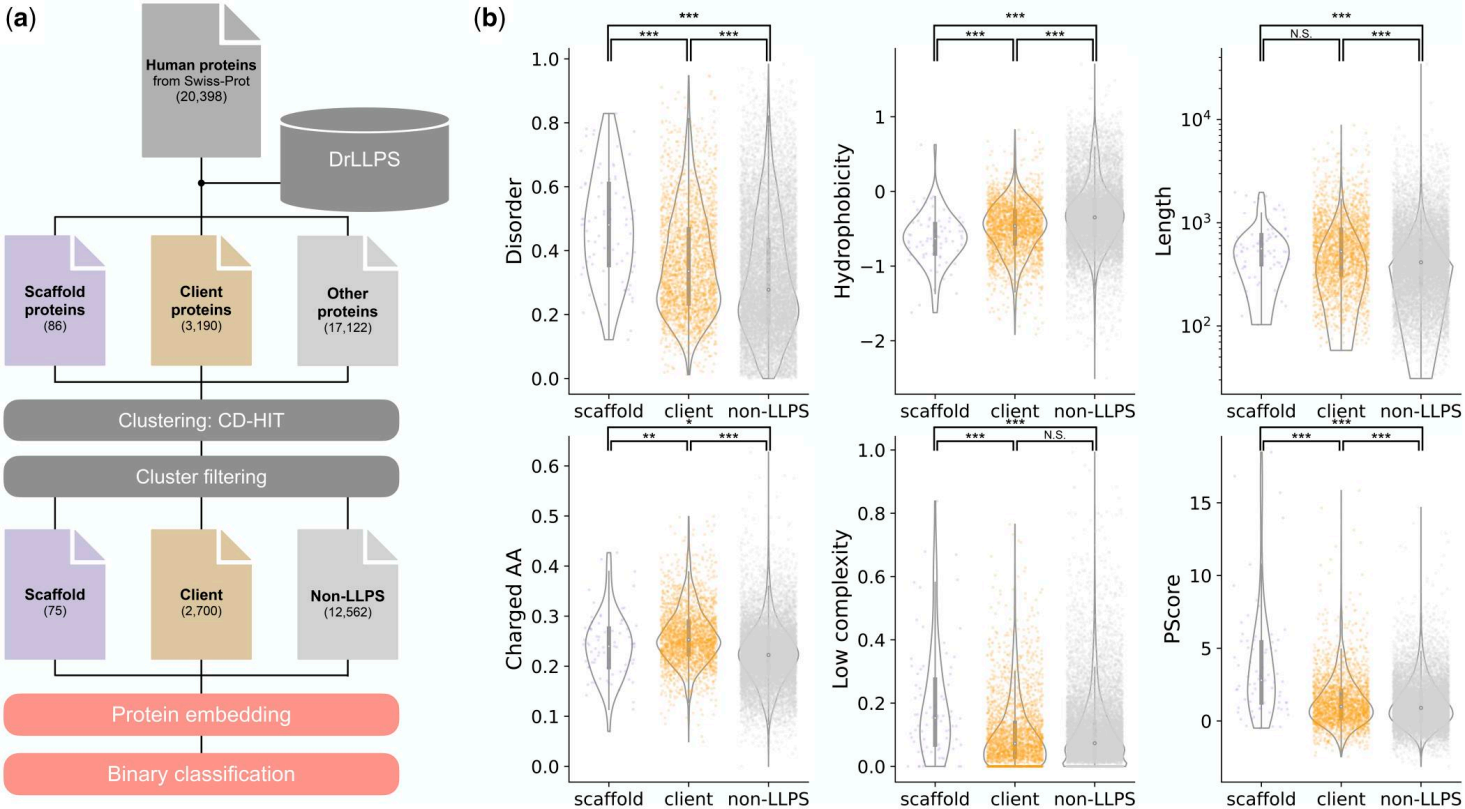
Workflow and amino-acid sequence properties. (a) Overall workflow. (b) Amino-acid sequence properties of scaffold, client, and non-LLPS proteins of *H.sapiens* after clustering. Each point corresponds to a representative protein. Symbols denote *P*-values of the Mann-Whitney U-test (two-sided). Non-significant (N.S.): *P *≥* *.05, *P <* *.05, **P <* *.01, ***P <* *.001.

A schematic figure of the overall framework is shown in Fig. 1a. The proteins in the two proteomes were clustered at a loose threshold of a 50% sequence identity to avoid redundancy as much as possible because similar sequences in both training and test datasets will make cross-validation give overfitted and erroneous results. If a cluster contained at least one scaffold or client protein, the longest among them was selected as its representative protein (see Supplementary Fig. S1). If a cluster contained neither a scaffold nor a client, the longest protein was selected and labeled as non-LLPS. If a cluster contained a regulator protein, the cluster was ignored in the subsequent analyses because it was not clear whether it is either scaffold, client, or non-LLPS.

For *H.sapiens*, the scaffold, client, and non-LLPS datasets contained 75, 2700, and 12 562 representative sequences, respectively, and for *S.cerevisiae*, 15, 573, and 5247 representative sequences, respectively (Supplementary Data S1).

### 3.2 Sequence property analysis of scaffolds and clients

Using the *H.sapiens* dataset, which contained more scaffold and client proteins than the *S.cerevisiae* dataset, we analyzed amino-acid sequence properties (Fig. 1b). Scaffolds, clients, and non-LLPS proteins showed the highest, intermediate, and lowest values, respectively, for ratios of intrinsically disordered regions (top left). This order was reversed for hydrophobicity (top middle). While the former result was consistent with previous reports on scaffold proteins, the latter was not because client proteins were assumed to have lower solvation energies for active transfer from the aqueous phase to condensates (Elbaum-Garfinkle *et al.* 2015, Molliex *et al.* 2015, Nott *et al.* 2015, Villegas and Levy 2021). We suppose that the low hydrophobicity of clients was because they had longer sequence lengths in our dataset (top right). Regardless of the trends, these results support an expectation that clients would have intermediate properties between scaffolds and non-LLPS proteins.

On the other hand, clients did not show values that were between those of scaffolds and non-LLPS proteins for ratios of charged amino acids (bottom left) and low complexity regions (bottom middle). These data suggest that clients are not simply “immature” scaffold proteins. It may also be notable that scaffolds did not show the highest value for the ratios of charged amino acids because this observation may be consistent with the assumption that client proteins have more charged amino acids.

Finally, clients did not show higher PScores than non-LLPS proteins (bottom right). PScore is the score from a scaffold predictor that considers π–π interactions, and its prediction software is available for download and stand-alone use (Vernon *et al.* 2018). Thus, a scaffold predictor is likely unable to predict clients, and more importantly, clients would have sequence properties different from those of scaffolds and be involved in LLPS through different molecular mechanisms. As discussed above, more electric charges and less low-complexity regions may characterize clients; however, no single characteristic showed apparent differences.

### 3.3 Language model-based accurate prediction of client proteins

To develop an accurate predictor of client proteins, we first used ProtTrans T5XLU50 (PT-T5XLU50) and ESM-2 3B (ESM2-3B) models to extract features from amino-acid sequences (Elnaggar *et al.* 2022, Lin *et al.* 2023). The vectors output from PT-T5XLU50 and ESM2-3B were then used as inputs for supervised machine learning: SVM, HGBC, RF, and NN (Cortes and Vapnik 1995, Breiman 2001, Ke *et al.* 2017). In addition, stacking ensemble learning that combines output from the four machine learning methods by logistic regression was used. When a binary classifier between two groups of proteins (e.g. clients and non-LLPS proteins) was trained and tested, the other group (e.g. scaffolds) was removed from the datasets. Due to imbalances in the numbers of client and non-LLPS proteins in the training datasets, downsampling by a random approach or Tomek links was also conducted (Tomek 1976). Scores were obtained by 5-fold cross-validation.

The results of the binary classification between clients and non-LLPS proteins of *H.sapiens* and *S.cerevisiae* are shown in Table 1. The four machine learning methods using PT-T5XLU50 vectors performed similarly and better than those using ESM2-3B vectors. Among the four machine learning methods, SVM trained without downsampling slightly outperformed the other methods for both datasets. In addition, the stacking ensemble learning slightly but significantly outperformed SVM (*P* < 0.01, Wilcoxon signed-rank test, Table 1). Finally, we adopted PT-T5XLU50 and the stacked model as our client predictor, naming it Seq2Phase.

**Table 1.** Scores of binary classifications between clients and non-LLPS proteins.^a^

Species	Embedder	ML method	ROC AUC	PR AUC	MCC
*H.sapiens*	PT-T5XLU50	SVM, n	0.855 ± 0.007	0.603 ± 0.022	0.483 ± 0.017
		HGBC, n	0.840 ± 0.011	0.566 ± 0.025	0.436 ± 0.028
		RF, T	0.843 ± 0.007	0.583 ± 0.026	0.457 ± 0.017
		NN, T	0.846 ± 0.008	0.590 ± 0.036	0.464 ± 0.033
	ESM2-3B	SVM, n	0.846 ± 0.008	0.565 ± 0.017	0.461 ± 0.015
		HGBC, n	0.824 ± 0.010	0.511 ± 0.021	0.374 ± 0.051
		RF, T	0.828 ± 0.013	0.534 ± 0.036	0.424 ± 0.022
		NN, n	0.819 ± 0.017	0.548 ± 0.043	0.410 ± 0.040
*S.cerevisiae*	PT-T5XLU50	SVM, n	0.853 ± 0.014	0.411 ± 0.035	0.388 ± 0.038
		HGBC, n	0.838 ± 0.017	0.358 ± 0.032	0.264 ± 0.046
		RF, n	0.843 ± 0.013	0.404 ± 0.018	0.332 ± 0.029
		NN, T	0.824 ± 0.031	0.382 ± 0.066	0.271 ± 0.063
	ESM2-3B	SVM, n	0.840 ± 0.019	0.388 ± 0.057	0.372 ± 0.024
		HGBC, n	0.817 ± 0.015	0.356 ± 0.024	0.177 ± 0.124
		RF, T	0.836 ± 0.013	0.387 ± 0.040	0.305 ± 0.039
		NN, n	0.802 ± 0.020	0.348 ± 0.027	0.232 ± 0.039

**Species**	**Embedder**	**ML method**	**ROC AUC**	**PR AUC**	**MCC**

*H.sapiens*	PT-T5XLU50	SVM	0.855 ± 0.012	0.618 ± 0.029	0.484 ± 0.021
		Ensemble	0.859 ± 0.012	0.630 ± 0.031	0.489 ± 0.030

a Area Under the Receiver Operating Characteristic Curve (ROC AUC), Area Under the Precision-Recall Curve (PR AUC), and Matthews Correlation Coefficient (MCC) are shown for each of *H.sapiens* and *S.cerevisiae* and for each embedding and machine-learning method. The best value based on the two downsampling methods and no downsampling is shown (r, T, and n represent random, Tomek link, and no downsampling, respectively). The mean and standard deviation for the cross-validation are shown.

**Figure 2.**
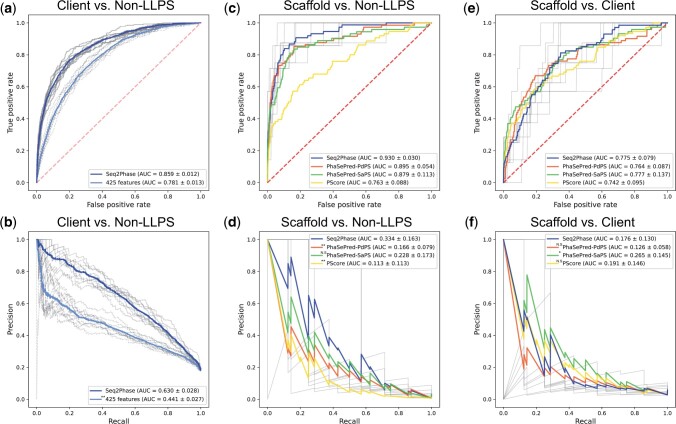
ROC curves and PR curves for LLPS-related protein prediction by Seq2Phase and existing methods. (a and b) The ROC and PR curves for client prediction of *H.sapiens* by Seq2Phase, 425 features are shown as blue and light blue lines, respectively. (c–f) The ROC and PR curves for scaffold prediction of *H.sapiens* by Seq2Phase, PhaSePred PdPS, SaPS, and PScore are shown as blue, orange, green, and yellow lines, respectively. For Seq2Phase, the curves for each of the 10-fold cross-validation are shown in gray, and the average curve is shown in blue. The dashed gray line is the diagonal line. Symbols indicate *P*-values of the Wilcoxon signed-rank test (two-sided) against Seq2Phase (N.S.: *q* ≥ 0.05, *q < 0.05, **q < 0.01).

Because there was no existing predictor of client proteins, we compared Seq2Phase with a baseline predictor that used naive 425 features that are known to be important for LLPS scaffolds: hydrophobicity, the sequence length, and ratios of amino acids, amino-acid dimers, intrinsically disordered regions, low-complexity regions, and charged amino acids. We compared the performances of the stacked model trained on these features and Seq2Phase. The results showed that Seq2Phase achieved a significantly higher performance (*P *<* *0.01, Wilcoxon signed-rank test), suggesting that Seq2Phase captures client characteristics that are not explicitly recognized yet (Fig. 2a and b).

Additionally, we compared the client protein prediction performance of Seq2Phase with that of existing scaffold predictors, PScore and PhaSePred. PhaSePred is a tool that extracts ten different features from an amino acid sequence and uses machine learning to predict scaffold (SaPS) and co-scaffold (PdPS) proteins (co-scaffolds are proteins that require other partners for condensate formation and are not clients) (Chen *et al.* 2022). The results showed that Seq2Phase achieved the highest performance (*P *<* *0.01, Wilcoxon signed-rank test), followed by PhaSePred PdPS, PhaSePred SaPS, and PScore (Supplementary Fig. S2). This result suggests that Seq2Phase captures the properties of clients that are not used by conventional methods.

To confirm that the same combination of PT-T5XLU50 and the stacked model can also predict scaffolds, we trained the models for the binary classification of scaffolds versus non-LLPS and scaffolds versus clients, and compared them to the existing scaffold predictors. Because the number of scaffolds was much smaller than the size of the PT-T5XLU50-embedded vector, we performed dimensionality reduction. Through PCA, we optimized the dimensionality and hyperparameters for individual models of the stacked model. The results indicated that, for the classification of scaffolds versus non-LLPS proteins, the optimal dimensions were 128 for NN, RF, and SVM and 64 for HGBC (Supplementary Fig. S3). For the classification of scaffolds versus clients, the optimal dimensions were 128 for RF and NN, 64 for HGBC, and 32 for SVM. Models input with those optimized dimensions were subsequently stacked and used for ensemble learning. For scaffolds versus non-LLPS, our method outperformed PScore and PhaSePred-PdPS and was comparable to PhaSePred-SaPS (Fig. 2c and d). For scaffolds versus clients, our method, PhaSePred-PdPS, and PScore performed equally and PhaSePred-SaPS outperformed them (Fig. 2e and f). Overall, the combination of PT-T5XLU50 and the stacked model can predict both client and scaffold proteins.

### 3.4 Consistency of Seq2Phase predictions with biological knowledge

We noted that our client datasets based on DrLLPS must not be complete because LLPS research is still in its infancy. Most importantly, the non-LLPS proteins in our datasets may actually contain condensate clients that have not been experimentally identified yet. That is, a part of “false positive” predictions by Seq2Phase could actually be “true positive” predictions. To examine this possibility, we checked the characteristics of proteins in the *H.sapiens* non-LLPS dataset that were predicted to be clients by Seq2Phase.

Seq2Phase predicted 2097 proteins to be clients in 12 562 non-LLPS proteins (Supplementary Data S2). We examined if Gene Ontology’s cellular component (GO-CC) terms of those 2097 “predicted clients” significantly contained 46 terms associated with membraneless organelles in PhaSepPro (32, 33, 34). The degree of enrichment was compared with that of “known clients” in DrLLPS by Fisher’s exact test with multiple testing corrections by the Benjamini–Hochberg method (*q*-value < 0.05) (Fisher 1935, Benjamini and Hochberg 1995).

**Figure 3.**
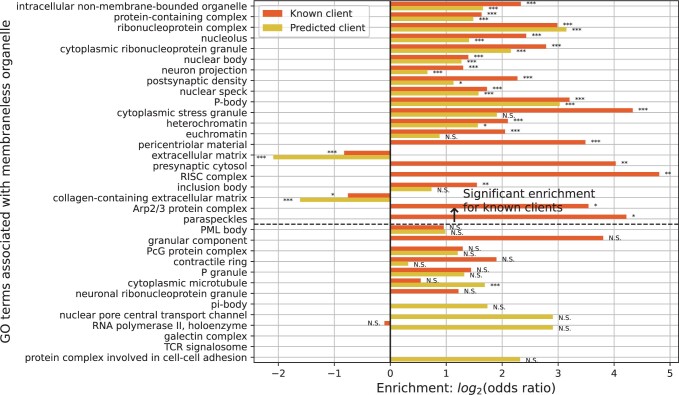
GO enrichment analysis of known and predicted clients. On the vertical axis, GO terms are listed in the order of decreasing *q*-values in the analysis of known clients. The *q*-values corrected for multiple testing of the Fisher’s exact test are shown by symbols (N.S.: *q* ≥ 0.05, *q < 0.05, **q < 0.01, ***q < 0.001). The absence of a bar means that there were no known or predicted clients with that GO.

As expected, the known and predicted clients were similarly enriched in most GO-CC terms associated with LLPS (Fig. 3; only 34 terms are shown because the non-LLPS proteins did not contain the other 12 terms). These similar enrichment patterns strongly suggested that predicted clients likely contained many true client proteins, and the “true” ROC AUC of Seq2Phase would be larger than 0.86. Known clients were positively enriched in 21 terms, and 11 among them showed significant enrichment for the predicted clients. *Intracellular non-membrane-bounded organelle* and *cytoplasmic ribonucleoprotein granule* are ancestral terms for many membraneless-organelle GO-CC terms such as *nucleolus* and *P-body* (Brangwynne *et al.* 2011, Kroschwald *et al.* 2015).

### 3.5 Visual inspection of sequence properties of clients and scaffold

**Figure 4.**
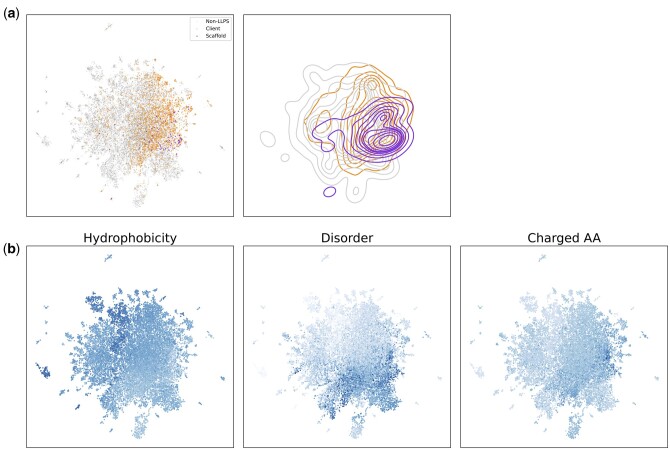
PT-T5XLU50-embedded vectors of *H.sapiens* protein sequences. 2D distributions of PT-T5XLU50-embedded vectors of *H.sapiens* protein sequences by UMAP are shown. (a) The scaffolds, known clients, and non-LLPS proteins are shown in purple, orange, and gray. The right panel shows the density distributions. (b) The left, middle, and right panels show the proportions of intrinsically disordered regions, hydrophobicity, and the proportion of charged amino acids on the same UMAP distribution.

To investigate why the proposed method can predict clients successfully, PT-T5XLU50-embedded vectors of the *H.sapiens* protein sequences were visualized using UMAP (McInnes *et al.* 2018). Their distributions of the scaffolds, known and predicted clients, and non-LLPS proteins are shown in Fig. 4a. Each group of the clients and scaffolds is mapped closely in this figure, showing that the PT-T5XLU50-embedded vectors capture their characteristics. In addition, the distributions of the clients and scaffolds overlap only partially; specifically, clients include scaffolds inside. Whereas most scaffolds may be like clients in regard to sequence properties, many clients (especially those on the upper right area) would not be like scaffolds.

Figure 4b visualizes the proportions of intrinsically disordered regions, hydrophobicity, and the proportion of charged amino acids on the UMAP representation. The client proteins are distributed to regions with different values of disordered regions, hydrophobicity, and the proportion of charged amino acids, showing that client proteins exhibit diverse sequence characteristics. This challenges the traditional textbook understanding that proteins forming condensates predominantly possess large intrinsically disordered regions. Our findings suggest a need to revise this view in light of the diverse characteristics observed in the LLPS-related proteins.

### 3.6 Inter-species client prediction by Seq2Phase

We then investigated if Seq2Phase trained on *H.sapiens* proteins can predict clients of *S.cerevisiae*, *M.musculus*, and *A.thaliana*. If this is the case, Seq2Phase is mainly based on physicochemical properties of client proteins and will be applicable to any newly sequenced genomes. We prepared *M.musculus* and *A.thaliana* data using Swiss-Prot and DrLLPS.

**Figure 5.**
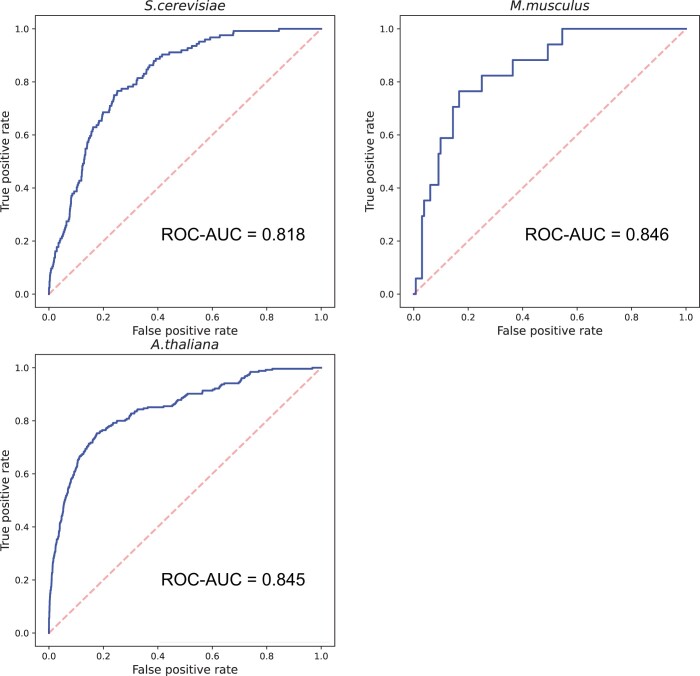
Inter-species client prediction by Seq2Phase. The AUC curves for client prediction of *S.cerevisiae*, *M.musculus*, and *A.thaliana* after the removal of protein sequences similar to those of *H.sapiens* are shown. Seq2Phase was trained using the *H.sapiens* dataset. The dashed gray line is the diagonal line.

The *S.cerevisiae*, *M.musculus*, and *A.thaliana* datasets contained 573, 1382, and 1149 client proteins, respectively. If we removed protein sequences similar to those of *H.sapiens* to avoid overestimation, the numbers of client sequences became 124, 17, and 255, respectively (Note that *M.musculus* is closely related to *H.sapiens* and they share many similar proteins). The inter-species validation based on those non-homologous proteins showed that Seq2Phase is not overfitted to *H.sapiens* and has broad applicability (Fig. 5). This also suggests what makes clients are general physicochemical features rather than specific interactions with scaffold proteins.

Finally, we applied Seq2Phase to the proteomes of *S.cerevisiae*, *M.musculus*, and *A.thaliana*. By excluding the client proteins annotated in DrLLPS, Seq2Phase predicted additional 1121, 4440, and 2944 client proteins, respectively. Therefore, we predict that more than hundreds or thousands of LLPS client proteins remain undiscovered in each species and that Seq2Phase will advance our understanding of still enigmatic molecular and physiological bases of LLPS as well as its roles in disease.

### 3.7 Guideline to interpret Seq2Phase scores

To provide a guess on how to interpret the prediction scores of Seq2Phase, we examined their distributions regarding clients, scaffolds, and non-LLPS. Here, the client model was trained using the client and non-LLPS data. The scaffold model was also trained using the scaffold, client, and non-LLPS data, where the clients were regarded not to be scaffolds.

**Figure 6.**
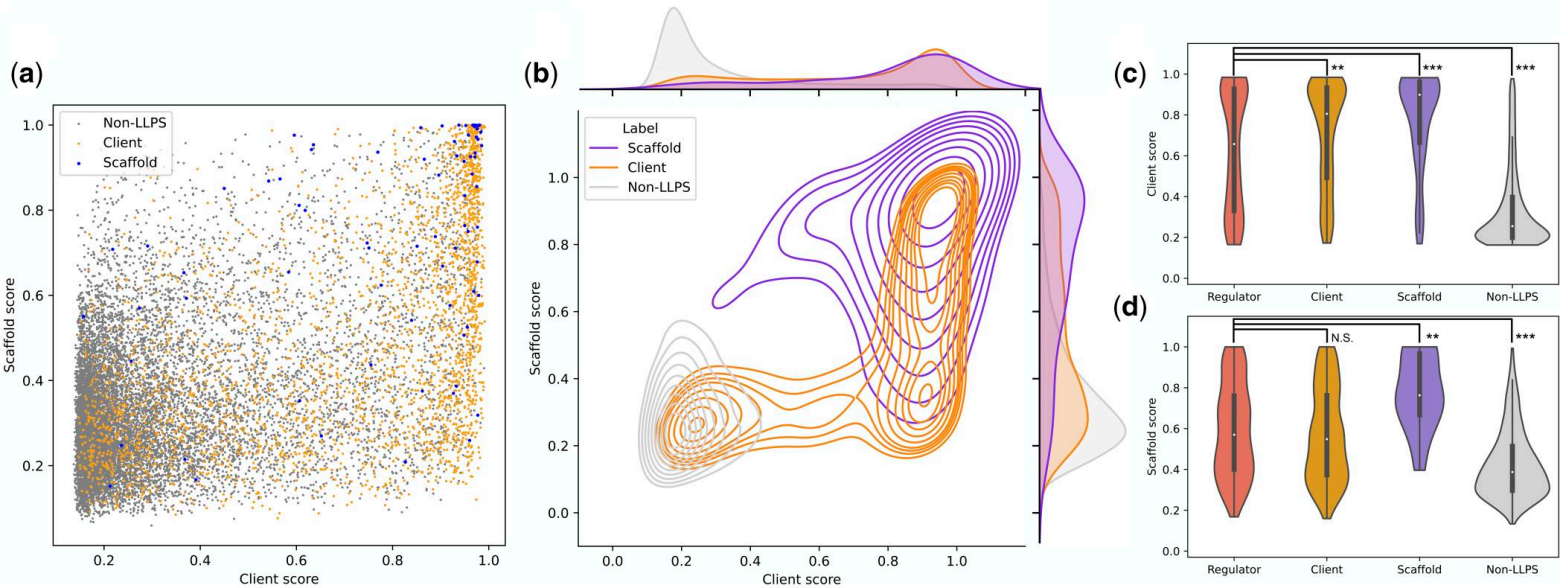
Distribution of prediction scores. (a) A plot of client (*x*-axis) versus scaffold scores (*y*-axis) predicted by Seq2Phase for human proteins after the clustering. (b) Density distributions of scores illustrated by seaborn.kdeplot. The distributions of the client and scaffold scores are shown above and to the right, respectively. (c and d) Distributions of the client and scaffold scores including those of regulator proteins. Symbols denote *P*-values of Mann–Whitney *U*-tests (two-sided): Non-significant (N.S.): *P* ≥ .05, **P < .01, ***P < .001.

The 5-fold cross-validation on the human proteome showed score distributions shown in Fig. 6a and b. While non-LLPS proteins had small client and scaffold scores and clients had large client scores, scaffolds tended to have large scaffold and client scores. This result is consistent with the observation that the PT-T5XLU50-embedded vectors of scaffolds were distributed in the area of the client vectors (Fig. 4). Thus, in practical applications, users would refer to the scaffold and client scores and classify proteins that were predicted to both categories as scaffolds.

We also evaluated the distribution of the Seq2Phase scores of LLPS-regulator proteins (Fig. 6c and d). The regulators exhibited significantly larger client scores than non-LLPS proteins, while their scaffold scores were comparable to those of clients. This implies that most regulators are client proteins and shows the importance of the prediction of client proteins to understand LLPS regulation.

### 3.8 Structural insight into client scores

**Figure 7.**
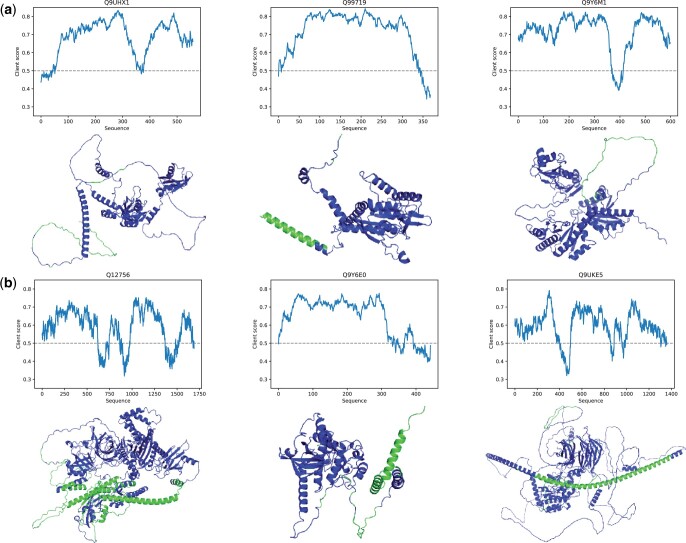
Region-wise client scores and predicted structures of proteins with the largest client scores. (a) Region-wise client scores and AlphaFold predicted structures of the top three client-like proteins from the client category (UniProt IDs: Q9UHX1, Q99719, and Q9Y6M1/Gene names: PUF60, SEPTIN5, and IGF2BP2). In the plots, the *x*-axis represents amino-acid sequences, and the *y*-axis denotes the client scores. In the ribbon structures, the blue and green regions indicate protein regions with large and small client scores, respectively. (b) Those of the top three cline-like proteins from the non-LLPS category (UniProt IDs: Q12756, Q9Y6E0, and Q9UKE5/Gene names: KIF1A, STK24, and TNIK).

It should be noted that Seq2Phase can compute client scores in a region-wise manner within each protein for investigating the amino-acid and structural context of client proteins. We trained Seq2Phase by four-fifths of known human clients and an equivalent number of non-LLPS to predict the client scores of the remaining proteins. From each of the client and non-LLPS test datasets, we selected the top 20 proteins with the highest client scores. Subsequently, we identified three proteins that contained many non-client regions and used them for further analysis. We visualized their client scores along the protein sequences with AlphaFold predicted structures (obtained from AlphaFold DB on 14 November 2023) (Fig. 7).

Surprisingly, many protein regions with large client scores were predicted to be structured regions (blue regions in Fig. 7), because preceding scaffold studies suggested that LLPS-related proteins contain intrinsically disordered regions. Remarkably, the RNA-recognition motifs in PUF60 and IGF2BP2 are RNA-binding domains and likely associated with localization to the RNP granules, which are recognized as membraneless organelles. Because Seq2Phase requires sequence data only and it is still technically difficult and time-consuming to experimentally determine protein regions responsible for LLPS, we believe that Seq2Phase will greatly help us understand the domain and structural context of LLPS biology.

## Supplementary Material

vbad189_Supplementary_Data
