# Supplementary material for: Seq2Phase: language model-based accurate prediction of client proteins in liquid–liquid phase separation
Source: Bioinform Adv. 2023 Dec 22;4(1):vbad189. doi: 10.1093/bioadv/vbad189 (PMC10777356; doi:10.1093/bioadv/vbad189)
Supplement: vbad189_Supplementary_Data [file vbad189_supplementary_data.zip › Supplementary_Fig.pdf]

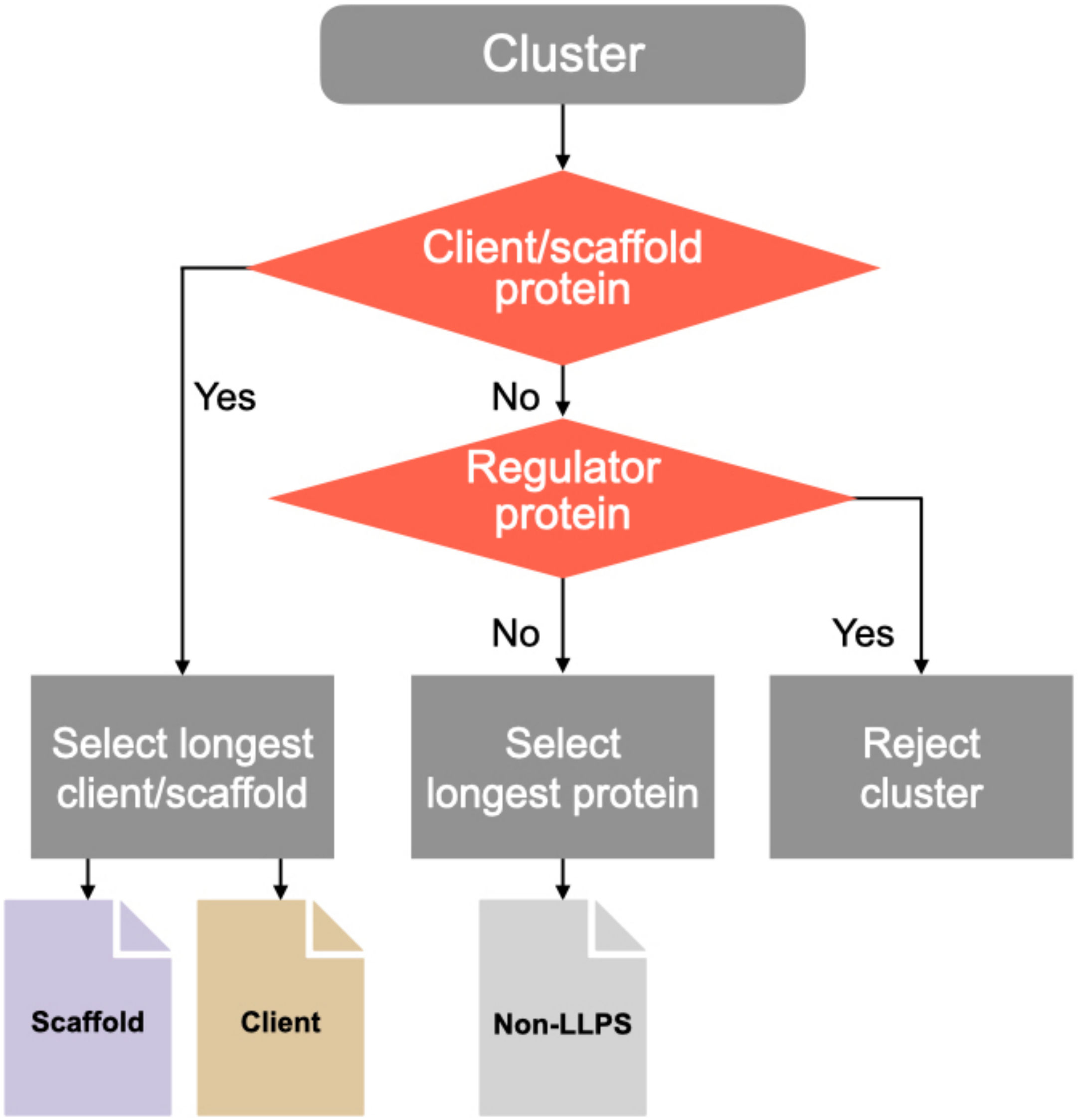

Fig. S1 Workflow of the cluster filtering. Workflow for selecting proteins from a cluster to build a dataset.

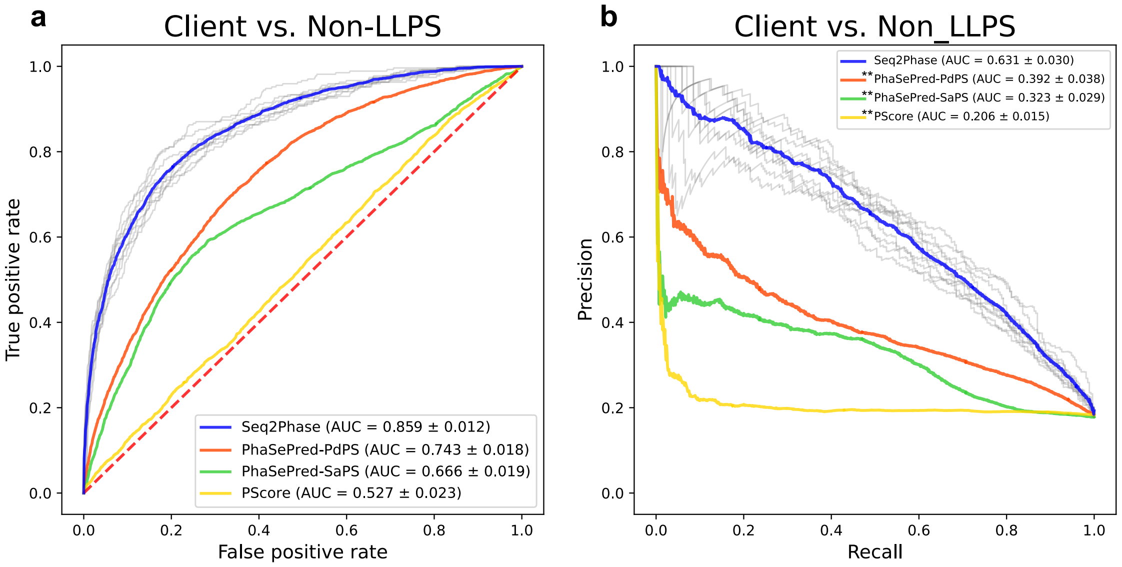

Fig. S2 ROC curves and PR curves for client prediction by Seq2Phase and existing methods. The ROC and PR curves for client prediction of H. sapiens by Seq2Phase, PhaSePred PdPS, SaPS, and PScore are shown as blue, orange, green, yellow lines, respectively. For Seq2Phase, the curves for each of the ten-fold cross validation are shown in gray, and the average curve is shown in blue. The dashed gray line is the diagonal line. Symbols indicate p-values of Wilcoxon signed-rank test (two-sided) against Seq2Phase (**: $q<0.01$).

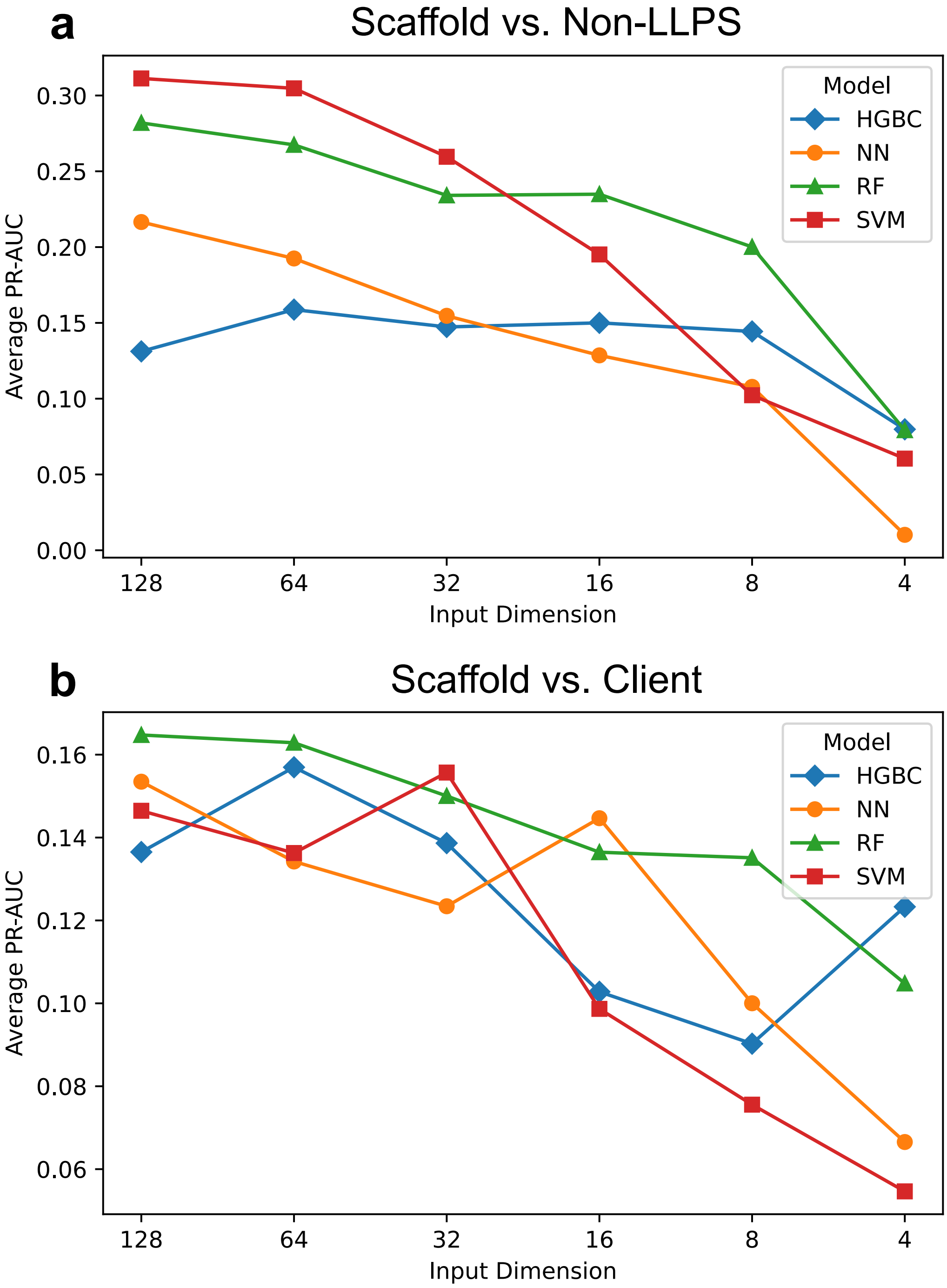

Fig. S3 PR AUC for scaffold prediction with dimensionality reduction. PR AUC for scaffold prediction using four ML models with dimensionality reduction by Principal component analysis (PCA).
